# Prevalence and determinants of wife-beating in Bangladesh: evidence from a nationwide survey

**DOI:** 10.1186/s12888-021-03652-x

**Published:** 2022-01-04

**Authors:** Md. Moyazzem Hossain, Faruq Abdulla, Azizur Rahman, Hafiz T. A. Khan

**Affiliations:** 1 Department of Statistics, Jahangirnagar University, Savar, Dhaka, Bangladesh; 2 Department of Applied Health and Nutrition, RTM Al-Kabir Technical University, Sylhet, Bangladesh; 3 School of Computing, Mathematics and Engineering, Charles Sturt University, Wagga Wagga, NSW 2678 Australia; 4 Public Health & Statistics, College of Nursing, Midwifery and Healthcare, University of West London, Brentford, UK

**Keywords:** Wife-beating, Ordinal logistics regression, Urban–rural settings, Bangladesh

## Abstract

**Background:**

Intimate partner violence (IPV) is a global public health concern, with women in low- and middle-income countries (LMICs) bearing a disproportionately high burden. This study investigates the prevalence and factors correlated with attitudes regarding wife-beating among Bangladeshi women in urban–rural contexts.

**Methods:**

A sample of 13,033 urban women and 51,344 rural women data from the Bangladesh Multiple Indicator Cluster Survey (MICS) 2019 were analyzed using the Chi-square test and ordinal logistic regression model.

**Results:**

The findings reveal that arguing with her husband is the widespread reason for wife-beating in Bangladesh (urban: 17.3%, rural: 21.9%), followed by neglecting the children (urban: 12.7%, rural: 15.8%). About 8% of urban women and 10% of rural women favoured the opinion that refusing to involve sexual intercourse is a legitimate justification for wife-beating. In comparison, around 5% feel that a husband has a right to beat his wife due to burning food. The respondents’ age, education, marital status, number of children, socioeconomic level, any health or physical difficulty, having problems becoming pregnant, and the husband’s age are all significant factors in justifying wife-beating.

**Conclusions:**

Bangladesh has a massive challenge in eliminating IPV. Women from lower socioeconomic classes, low levels of education, other challenges, and residents of rural areas are particularly more vulnerable than their urban counterparts. Therefore, it is vital to develop a proper action plan that considers women’s education and occupation to raise awareness of the various implications of wife-beating in women, particularly in Bangladesh’s rural areas.

## Background

Violence against women is omnipresent. Various terms have been used for violence against women in past literature, such as domestic violence (DV) or intimate partner violence (IPV). IPV is a pattern of coercive tactics used by one person against an intimate partner, including but not limited to psychological, physical, social, emotional, sexual, and economic mistreatment, usually to gain and maintain power and control [1]. An intimate partner or ex-partner’s physical, sexual, or psychological injury causes IPV, including physical assault, sexual harassment, psychological abuse, and perceived behavioural abuse [2]. Over the last few decades, the number of studies examining violence against women has risen considerably, and violence against women has progressively become a public health concern [3–5]. About one in every three women in the world is reported to have experienced intimate partner abuse at some point in their life, though numbers vary significantly by country [2, 5]. Lifetime intimate partner violence prevalence estimates range from 20% in the Western Pacific, 22% in high-income countries and Europe, and 25% in the world health organization (WHO) Americas Regions, to 33% in the WHO African region, 31% in the WHO Eastern Mediterranean region, and 33% in the WHO South-East Asia region [2]. The lifetime prevalence of IPV was reported at about 42% and 30% in South Asia and worldwide, respectively [6, 7]. The previous research reveals 4% to 10% of the respondents’ opinion that their wives had run away from home because of DV, and 3% pointed out that their wives had ever sought medical treatment for their injuries in India [8, 9]. In addition, children who were exposed to violence in their families of origin, either through childhood violence or watching the interparental conflict, were more likely to employ violence in their families as adults than children who were never exposed to family violence [2, 10].

Moreover, the research explores several social and demographic determinants, including age at first marriage, spousal age difference, marriage type, education, consumption of alcohol, and household characteristics. Some of these predictors have been identified to be inconsistently correlated with Intimate partner violence (IPV) in some cases [11–14]. For women, physical domestic violence has several ramifications. Induced abortion, HIV, depression, suicide, and other mental health issues such as anxiety, phobias, irritable bowel syndrome, chronic fear, cardiac problems, and gynaecological issues are more common among victims [15–17]. Previous research pointed out that IPV victims are more likely to have unwanted pregnancies, have numerous abortions, and have less sexual autonomy [18]. Moreover, women subjected to physical domestic violence are more likely to have low-birth-weight kids, unintended pregnancies, miscarriages, and abortions, as well as sexually transmitted diseases [15, 19–21].

Domestic violence in developing nations has risen from relative obscurity to a major source of worry among a growing group of experts and policymakers concerned with women’s health and status over the last decade [22, 23]. Domestic violence, especially in low-income countries, is a public health and gender-based issue [24] and increases the risk of depression among non-pregnant women [25]. Young age, poverty, inadequate education, early marriage, suffering mother-to-father abuse in the home, childhood violence, dowry, and poor spousal communication are critical factors of IPV in Bangladesh [26–29]. In many developing nations, including Bangladesh, there is widespread acceptability of ‘wife-beating,’ a typical form of IPV, frequently perpetrated by societal conventions and gender roles [30]. A study pointed out that spousal violence against women is frequent in Bangladesh [31]. In some societies, traditional beliefs condoning wife-beating are used to justify IPV against women. In contrast, marital traditions have been related to sexual violence, such as paying bridewealth, which culturally gives the husband sexual rights over the wife [32–34]. It’s been reported that wife-beating is linked to patriarchal institutional domination, unequal gender power distribution, household resource control, sociodemographic characteristics and other household activities [33, 35–40]. However, a woman who believes wife-beating to be “unjustifiable” is more likely to recognize her increased sense of entitlement, self-esteem, and status, as well as to reflect positively on her sense of empowerment [41].

Many questions about the prevalence of IPV remain unanswered. Some significant factors, such as the indifferent conduct of women, husband arguments, neglecting children, less desire to have sex, etc., are not considered in the existing research in Bangladesh and based on the most recently available data. The rationale for wife-beating shows that a woman generally accepts a man”s authority to control her preferences, including violence. As a result, it is critical to understand the scope and causes for wife-beating acceptance in low-income countries, especially Bangladesh. The specific factors linked with the justification and acceptance of wife-beating in Bangladesh are needed to be examined. This study, therefore, aims to examine the relationship between socioeconomic attributes of women aged 15 to 49 years and their reasoning for wife-beating in urban–rural settings of Bangladesh.

## Methods

### Data

This study used the secondary dataset of women from the Multiple Indicator Cluster Survey (MICS)-2019, a nationwide survey conducted in Bangladesh funded by UNICEF [42]. The data include information on public health-related indicators of the eight geographical divisions of Bangladesh. The MICS applied a two-stage stratified sampling procedure for collecting the required information. The districts were used as the primary sampling strata, and many census enumeration areas (EAs) were carefully sampled using the probability relative to the measure inside each stratum. Following the family unit posting within the chosen EAs, a systematic sample of 20 families was taken from each EA. The number of sampled households in the survey was 64,400 in 2019. However, this study included currently married and ever-married women; incomplete cases and never-married women were excluded. Therefore, the subsequent analysis of this study is based on *N* = 64,378 women in 2019.

### Outcome variable

The outcome variable is the level of violence, which is calculated from any five of the reasons such as “goes out without telling husband”; “neglects the children”; “argues with husband”; “refuses sex with husband”; “burns the food” among women aged 15–49 years in Bangladesh. For each case, a score “0” is assigned if a respondent thought a beating would be justified, and “1” if a respondent thought a beating would not be justified. Then adding the score of these five variables (min = 0 and max = 5) and the level of the target variable is categorized as:

- None = no violence occur due to above-mentioned reasons (0);
- Mild = if they suffer from any two of them (1–2);
- Moderate = if they suffer from any three of them (3); and
- Severe = if they suffer from any four or more of them (4–5).

### Predictor variables

This study aims to identify the association between violence and several socio-economic and demographic variables. The independent variables included in the analysis follow: the age of women (15–19, 20–24, 25–29, 30–34, 35–39, 40–44, and 45–49 years), educational status (Pre-primary or none, Primary, Secondary and Higher secondary +); geographical division (Barishal, Chattogram, Dhaka, Khulna, Mymensingh, Rajshahi, Rangpur, and Sylhet); having functional difficulties (Yes, No); ethnicity of household head (Bengali, Others (Chakma, Santal, Marma, Tripura, and Garo)); the age of husband (15–21, 22–25, 26–30, 31–35, 36–40, 41–45, and 46 + years); wealth index (poorest, poorer, middle, richer, and richest); husband has more than one wives (Yes, No); the number of ever born children (none, 1–2, 3–4, 5–12); wanted the last child (Yes, No) and able to get pregnant (Yes, No). The variable selection was motivated by the availability in the MICS-2019 dataset and self-efficacy and guided by relevant literature.

### Statistical analysis

The summary statistics were calculated for categorical variables, and the Chi-square test was also performed for assessing the primary association between the various forms of intimate partner violence and other categorical variables. Finally, as the outcome variable is classified according to their order of magnitude, Ordinal Logistic Regression (OLR) analysis has been conducted to assess the relationship between the level of violence and socio-demographic factors. Suppose, $Y$ represent an ordinal outcome in conjunction with j categories, then $P(Y\le j)$ is the cumulative probability of $Y$ less than or equal to a specific category $j=\mathrm{1,2},\dots ,J-1$. The odds of being less than or equal to a specific category can be described as, $\frac{P\left(Y\le j\right)}{P\left(Y>j\right)}$ for $j=\mathrm{1,2},\dots ,J-1$ since for $P\left(Y>J\right)=0$ is undefined. The log odds are also recognized as the logit, so that $\mathrm{log}\frac{P(Y\le j)}{P(Y>j)}=logit(P(Y\le j)$. The general form of the OLR model can be written as,

$$logit\left(P\left(Y\le j\right)\right)={\theta }_{j}-\left[{\beta }_{1}{X}_{i1}+{\beta }_{2}{X}_{i2}+\cdots +{\beta }_{p}{X}_{ip}\right]$$

where, $logit( )$ is the link function, ${\theta }_{j}$ is the threshold for the $j$ th category, ${X}_{i1},{X}_{i2},\dots ,{X}_{ip}$ are the values of the predictors for the $i$ th case, ${\beta }_{1},{\beta }_{2},\dots ,{\beta }_{p}$ are regression coefficients, and $p$ is the number of regression coefficients [43–46].

In an Ordinal Logistic Regression (OLR), the negative log–log $-\mathrm{log}(-\mathrm{log}\left(\mathrm{y}\right)))$ has been used in this study since lower categories are more likely than others to build up the OLR model [47, 48]. For better understanding and interpretations odds ratio (OR) has also been calculated. All the analysis was performed using IBM SPSS 25.

## Results

**Fig. 1:**
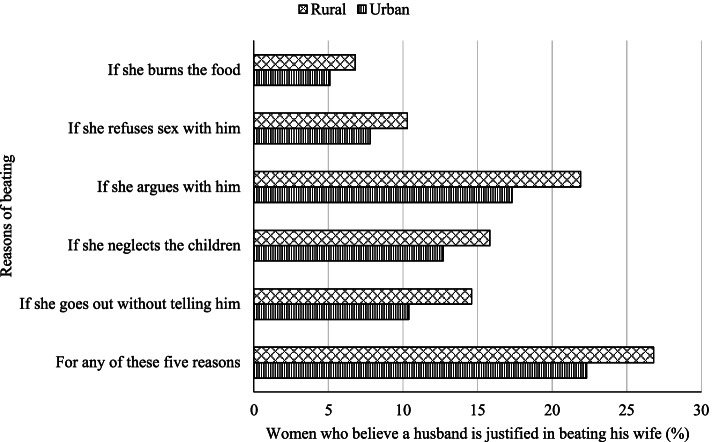
Reasons of beating women by their partners

Figure 1 depicts the prevalence of the outcome variable in urban–rural settings, which is the age of women who believe they would justify wife-beating in a specific situation by their husband. It is seen that among the mentioned five reasons, the least prevalence is observed among women who say that it is justified to beat them by their husbands if they burn food (rural: 6.8%, urban: 5.1%). The heights prevalence of wife-beating has occurred against women who argue with their husbands. Thus, more violence is happening in rural areas than urban areas in Bangladesh. Moreover, it is observed that more than 25% of violence (in any form) happened against women in rural areas, while in urban areas, it is just above 20% (Fig. 1).

Table 1 represents the results of women’s justification of wife-beating according to sociodemographic characteristics in urban and rural settings. Results depict that women’s age is a significant factor in violence against women in urban and rural regions. Less violence is observed among women whose age is less than 30 years compared to older women. Education is also significant for violence against women. Women having secondary or higher education were less likely to justify wife-beating than illiterate or primary educated women. Women who have some functional difficulties and cannot get pregnant are frequently justified wife-beating than their counterparts.

**Table 1:** Distribution of IPV, and associations with selected variables of women aged 15–49 years, MICS 2019

Characteristics	Women (15–49 years) who believe a husband is justified in beating his wife (%)											
	**Urban (*n* = 13,033)**						**Rural (*n* = 51,344)**					
	If she goes out without telling him	If she neglects the children	If she argues with him	If she refuses sex with him	If she burns the food	For any of these five reasons	If she goes out without telling him	If she neglects the children	If she argues with him	If she refuses sex with him	If she burns the food	For any of these five reasons
**Age**												
15–19	6.9	8.4	11.8	6.7	3.8	16.8	10.2	11	15.3	7.9	5.6	19.9
20–24	8.7	12.5	16.5	6.7	4.7	20.7	12.6	14	19.3	8.9	5.7	24.2
25–29	10.9	13.3	17.7	8	4.8	22.7	13.9	15.7	21.6	10.2	6.4	27
30–34	11.5	14.5	19.8	7.9	5.6	25.1	15.6	16.5	23.3	10.5	6.8	28.6
35–39	12.4	13.5	18.6	7.6	4.8	24.4	17.7	19.3	26	11.9	8.2	30.7
40–45	12.0	13.5	19.4	9.1	6.2	24.8	17.4	18.2	25.8	12.6	7.5	30.5
45–49	12.1	14.7	19.4	10.5	7	24	18.5	19.3	27	12.5	9.1	31.1
***p*-value**	< 0.001	< 0.001	< 0.001	< 0.001	< 0.001	< 0.001	< 0.001	< 0.001	< 0.001	< 0.001	< 0.001	< 0.001
**Education**												
Pre-primary or none	19.1	21.9	29.2	14.1	10.1	35.4	22.6	24.4	32.1	16.8	12.2	36.5
Primary	15.8	20.1	25.8	12.3	8.2	32.1	18.1	19.6	27.1	12.4	8.5	32.2
Secondary	9.7	11.3	16	6.9	4.1	21.2	12.6	13.4	19.2	8.6	5.3	24.1
Higher secondary +	3.6	5.3	7.6	3.1	2.1	11	5.6	6.6	9.7	4.4	2.6	14.2
***p*-value**	< 0.001	< 0.001	< 0.001	< 0.001	< 0.001	< 0.001	< 0.001	< 0.001	< 0.001	< 0.001	< 0.001	< 0.001
**Division**												
Barishal	8.8	10.4	14.2	6.5	2.6	20.5	12	12.8	17	7.9	2.8	25
Chattogram	10.7	11.5	16.3	7.8	4.4	21.4	13.8	13.9	20.4	10.7	6.3	25.3
Dhaka	9.4	14.4	17.5	7.5	5.7	22.7	14.4	16.9	22.6	8.3	6.8	27.5
Khulna	9.1	9.6	17	6.3	3.3	21.4	9.9	10.3	18	6.9	3.2	21.6
Mymensingh	10.6	12.7	20.9	11.2	9.6	26.4	8.8	11.5	21.7	11.3	8.4	24.2
Rajshahi	11.1	11.7	19.2	6.9	2.2	23.4	18.4	18.6	28.9	10.3	3.5	33.3
Rangpur	17.7	20.5	21.9	12	10.3	28.7	25.1	27.4	29.8	18.7	16.9	36.8
Sylhet	7.5	9	11.7	7.8	6.5	13.9	10.5	11.8	14.3	9.3	7.4	16.5
***p*-value**	< 0.001	< 0.001	< 0.001	< 0.001	< 0.001	< 0.001	< 0.001	< 0.001	< 0.001	< 0.001	< 0.001	< 0.001
**Functional Difficulties**												
Yes	16.8	15.9	23	12.4	8.4	29.5	20.4	21.5	28.8	13	8.5	35.3
No	10.6	13.1	17.9	7.8	5.1	22.8	15.1	16.3	22.6	10.6	6.9	27.4
***p*-value**	< 0.001	0.116	0.012	0.002	0.006	0.003	< 0.001	< 0.001	< 0.001	0.005	0.027	< 0.001
**Ethnicity of household head**												
Bengali	10.3	12.6	17.3	7.8	5	22.3	14.7	15.8	22.1	10.4	6.8	26.9
Other	11.9	15.9	15.9	9.1	8.3	23	11	14.1	16.9	8.4	7	20.7
***p*-value**	0.408	0.123	0.603	0.412	0.028	0.765	< 0.001	0.101	< 0.001	0.029	0.783	< 0.001
**Age of husband (Years)**												
** 15–21**	19.3	20.2	25.4	17.5	7.9	31.6	13.9	15.7	21.3	11.0	7.0	27.3
** 22–25**	14.3	17.9	21.4	11.2	6.9	26.3	14.6	16.5	22.0	10.4	6.1	26.7
** 26–30**	11.5	14.9	20.7	8.6	5.4	26.2	14.2	15.3	21.5	10.1	6.5	26.4
** 31–35**	10.2	13.6	18.4	6.9	4.8	22.5	14.0	15.5	21.4	10.0	5.9	26.4
** 36–40**	10.9	13.4	18.5	8.0	5.5	24.1	15.7	17.2	23.6	10.5	7.3	29.2
** 41–45**	11.7	14.3	18.6	7.4	4.5	24.0	16.2	17.5	24.7	11.3	7.0	29.6
** 46 + **	12.3	13.8	19.4	9.0	5.8	24.8	17.8	18.8	26.1	12.0	7.9	30.6
***p*-value**	0.02	0.09	0.23	< 0.001	0.27	0.10	< 0.001	< 0.001	< 0.001	< 0.001	< 0.001	< 0.001
**Wealth Index Quantile**												
Poorest	21.4	23.9	31.6	14.4	9.5	37.3	19.8	21.5	28.5	13.4	9.7	34
Second	17.6	19.4	26.7	10.9	8.3	32.4	16.7	18.7	25.8	12.3	8.5	30.6
Middle	12.7	15.4	20.2	9.4	6	26.2	13.3	14.2	20.4	9.7	6	25.5
Fourth	10.9	12.9	18.8	8.4	5.1	24.7	10.8	11.4	16.5	7.4	4.1	20.9
Richest	6.5	8.9	11.9	5.5	3.6	16	7.7	7.8	11.4	5.7	3.4	14.7
***p*-value**	< 0.001	< 0.001	< 0.001	< 0.001	< 0.001	< 0.001	< 0.001	< 0.001	< 0.001	< 0.001	< 0.001	< 0.001
**Husband has more wives**												
Yes	11.6	22.1	27.5	11.9	10.8	33.7	21.8	23.1	30.3	14.9	10.9	37.7
No	11.4	13.9	19.0	8.2	5.2	24.2	15.6	16.9	23.5	10.8	6.9	28.4
***p*-value**	< 0.001	< 0.001	< 0.001	0.015	< 0.001	< 0.001	< 0.001	< 0.001	< 0.001	< 0.001	< 0.001	< 0.001
**Number of ever born children**												
None	9.8	11.4	15.4	6.6	4.8	19.7	11.7	12.3	17.9	8.5	5.5	22.2
1–2	10.5	13.6	18.0	7.4	4.7	23.0	15.1	16.3	22.6	10.1	6.3	27.7
3–4	14.2	16.3	22.0	10.4	7.0	28.0	17.3	18.8	26.1	12.4	8.1	30.9
5–12	17.0	17.4	26.5	12.8	10.0	32.6	19.9	21.2	28.5	14.5	10.8	32.6
***p*-value**	< 0.001	< 0.001	< 0.001	< 0.001	< 0.001	< 0.001	< 0.001	< 0.001	< 0.001	< 0.001	< 0.001	< 0.001
**Wanted last child then**												
Yes	11.1	14.5	18.6	8.5	6.4	24.1	13.9	15.2	21.1	10.4	6.8	25.6
No	11.8	15.3	22.3	9.7	5.4	26.5	13.7	17.8	22.7	10.9	6.5	27.3
***p*-value**	0.72	0.70	0.10	0.48	0.48	0.32	0.85	0.008	0.145	0.59	0.68	0.13
**Able to get pregnant**												
Yes	11.2	13.0	18.3	7.6	5.1	22.9	14.7	15.1	21.4	9.6	6.0	26.4
No	13.7	16.0	20.7	8.2	5.8	27.3	17.6	18.8	25.9	10.9	6.5	30.7
***p*-value**	0.06	0.04	0.14	0.55	0.47	0.01	< 0.001	< 0.001	< 0.001	0.03	0.27	< 0.001

Moreover, women from higher socioeconomic classes would justify wife-beating less than women having lower socioeconomic status, i.e., wealth index is also a potential factor for violence against women in rural and urban settings. Furthermore, women with kids (0–2 children) justified wife-beating less frequently than women with three or more children. The age of women (28.7%) who reside in the Rangpur division justify wife-beating in any of the considered five reasons than women from other divisions in the country. However, females who resided in the Sylhet division less frequently justified wife-beating than women who lived in other divisions in both urban and rural settings (Table 1).

**Fig. 2:**
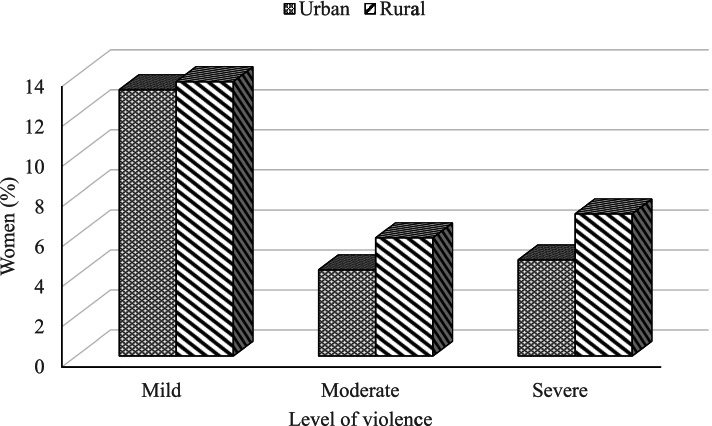
Level of violence against women

Figure 2 illustrates the level of violence observed against women. It is seen that a mild level of violence is more frequent than a moderate and severe level of violence in both urban and rural areas of Bangladesh. Interestingly, the prevalence of mild violence is just above 13% in rural and urban areas. About 5% of violence against women is a moderate level in urban and rural areas, though this percentage is lower in urban areas than rural areas. Approximately 5% of violence has happened in severe levels in urban areas, and more than 7% of severe violence occurred in rural areas against women in Bangladesh (Fig. 2).

The results of the ordinal logistic regression model are presented in Table 2. The target variable for this analysis is the levels of violence against women (mild, moderate, and severe). The findings depict that age is significantly linked with the justification of wife-beating in Bangladesh’s urban and rural areas. Results suggest that women with low educational qualifications were substantially more likely to justify wife-beating than women who complete secondary or higher education in urban and rural areas. In rural areas, females who are illiterate or have pre-primary education have a 2.37 times higher likelihood of justifying to be wife-beating than females whose educational qualification is higher secondary or above. Women who had functional difficulties were 1.45 and 1.22 times more likely to justify wife-beating than their counterparts in urban and rural areas. Women who lived in urban areas of Rajshahi (AOR: 2.42) and Chattogram (AOR: 2.2) division are more likely to justify wife-beating than women who lived in an urban area of Sylhet division. However, women who resided in rural areas of Rangpur (AOR: 3.1) and Khulna (AOR: 3.09) have more chance to justify of wife-beating than women who lived in rural areas Sylhet division. Comparatively, women who lived in the Barishal division are less likely to justify wife-beating. Women whose husband’s age is less than 46 years have less likelihood to justify wife-beating than women whose husband’s age is more than 46 years or more. Moreover, higher socioeconomic status was related to less justification of wife-beating in both urban and rural areas (Table 2).

**Table 2:** Results of ordinal logistic regression model for justifying wife-beating

Variable and their label	Urban			Rural		
	**Coefficient**	**95% CI**	**Adjusted OR**	**Coefficient**	**95% CI**	**Adjusted OR**
**Threshold (level of violence)**						
None	18.07	(15.89, 20.26)	-	4.74	(2.65, 6.83)	-
Mild	19.20	(17, 21.4)	-	5.55	(3.46, 7.64)	-
Moderate	19.85	(17.64, 22.06)	-	6.19	(4.1, 8.28)	-
**Age (years)**						
15–19	1.64	(1.46, 1.81)	5.14***	1.1	(-0.01, 2.21)	3.01*
20–24	1.26	(1.09, 1.42)	3.52***	0.93	(0.32, 1.54)	2.54**
25–29	1.12	(0.96, 1.29)	3.08***	0.99	(0.01, 1.98)	2.7**
30–34	1.19	(1.03, 1.35)	3.29***	1.05	(-0.15, 2.24)	2.85*
35–39	1.59	(1.43, 1.75)	4.91***	1.13	(0.54, 1.72)	3.1***
40–44	1.60	(1.45, 1.75)	4.95***	1.06	(0.14, 1.97)	2.88**
45–49 (Ref.)	-	-	-	-	-	-
**Education**						
Pre-primary or none	-0.01	(-0.9, 0.87)	0.99*	0.86	(0.49, 1.23)	2.37***
Primary	0.73	(0.11, 1.36)	2.08**	0.66	(0.35, 0.98)	1.94***
Secondary	0.03	(-0.54, 0.6)	1.03	0.27	(-0.01, 0.55)	1.32*
Higher secondary + (Ref.)	-	-	-	-	-	-
**Has functional difficulties**						
Yes	0.37	(-0.87, 1.62)	1.45*	0.2	(0, 0.4)	1.22**
No (Ref.)	-	-	-	-	-	-
**Number of children ever born**						
1–2	-0.10	(-1.17, 0.98)	0.91*	0.29	(0.1, 0.48)	1.34***
3–4	0.42	(-0.6, 1.43)	1.52*	0.2	(0, 0.4)	1.22**
5–12 (Ref.)	-	-	-	-	-	-
**Division**						
Barishal	-0.77	(-2.25, 0.71)	0.46	0.09	(0.04, 0.14)	1.09***
Chattogram	0.79	(-0.15, 1.73)	2.2*	1.01	(0.64, 1.38)	2.74***
Dhaka	0.22	(-0.76, 1.2)	1.25	0.89	(0.5, 1.28)	2.44***
Khulna	0.07	(-1.04, 1.18)	1.07	1.13	(0.72, 1.53)	3.09***
Mymenshing	0.70	(-0.68, 2.07)	2**	0.82	(0.36, 1.28)	2.27***
Rajshahi	0.88	(-0.15, 1.91)	2.42*	0.92	(0.5, 1.35)	2.52***
Rangpur	0.60	(-0.49, 1.69)	1.82	1.13	(0.72, 1.54)	3.1***
Sylhet (Ref.)	-	-	-	-	-	-
**Ethnicity of household head**						
Bengali	-0.04	(-1.06, 0.98)	0.96	0.71	(0.2, 1.22)	2.04**
Other (Ref.)	-	-	-	-	-	-
**Age of husband (years)**						
15–21	-0.65	(-2.06, 0.75)	0.52	-0.24	(-1.09, 0.61)	0.79
22–25	-0.15	(-0.3, -0.01)	0.85**	-0.25	(-0.49, 0)	0.78**
26–30	-0.18	(-0.59, 0.22)	0.83	-0.04	(-0.45, 0.38)	0.96
31–35	-0.02	(-0.04, 0)	0.98*	-0.12	(-0.24, -0.01)	0.88**
36–40	-0.08	(-0.18, 0.01)	0.92*	-0.18	(-0.38, 0.02)	0.83*
41–45	-0.06	(-0.16, 0.04)	0.94	-0.12	(-0.26, 0.02)	0.88*
46–90 (Ref.)	-	-	-	-	-	-
**Wanted last child then**						
Yes	-0.03	(-0.48, 0.43)	0.97	0.01	(-0.19, 0.21)	1.01
No (Ref.)	-	-	-	-	-	-
**Able to get pregnant**						
Yes	-0.28	(-0.67, 0.1)	0.75*	-0.02	(-0.04, 0)	0.98*
No (Ref.)	-	-	-	-	-	-
**Husband has more wives**						
Yes	-0.16	(-1.38, 1.06)	0.85**	0.1	(-0.01, 0.21)	1.11*
No (Ref.)	-	-	-	-	-	-
**Wealth index**						
Poorest	0.24	(-0.45, 0.94)	1.28**	0.87	(0.52, 1.22)	2.38***
Second	0.61	(-0.13, 1.35)	1.85*	0.45	(0.08, 0.81)	1.56**
Middle	0.36	(-0.24, 0.95)	1.43*	0.29	(0.07, 0.51)	1.33**
Fourth	0.47	(-0.02, 0.96)	1.6*	0.29	(0.01, 0.57)	1.34**
Richest (Ref.)	-	-	-	-	-	-

*Ref.* Reference category, *CI* Confidence interval, *, **, *** represents significant at 10%, 5% and 1% level respectively

## Discussion

Intimate partner violence, particularly wife-beating, is a persistent global health problem, especially in developing countries such as Bangladesh. Even though the entire world is working to improve women and children’s physical and mental health, wife-beating works against the entire globe by negatively impacting women’s and children’s health.. There are studies on this issue in Bangladesh; however, to the best of our knowledge, this is the first study to focus on the association between socioeconomic attributes of women aged 15–49 years and her justification of wife-beating in urban–rural settings of Bangladesh.

This study considers five reasons for the justification of wife-beating: “going out without telling husband”, “neglecting the children”, “arguing with husband”, “refusing sexual intercourse”, and “burning the food”. The findings revealed that arguing with her husband is the most common perceived reason for wife-beating in Bangladesh (urban: 17.3%, rural: 21.9%), followed by neglecting the children (urban: 12.7%, rural: 15.8%). The reason behind this finding may be the loss of temperament, and husbands, along with some women and their community, believe that partners would beat their wives if they did something wrong [49]. The urban–rural difference may be the consequence of more remarkable socio-economic development in urban compared to rural areas. Moreover, in rural and urban areas, roughly 10% and 8% of women agree that refusing to have sexual intercourse is a legitimate justification for wife-beating, respectively. In comparison, more than 5% (urban: 5.1%, rural: 6.8%) feel that a husband has the right of beating his wife for the reason of burning food. These results are consistent with the results of another study [50]. Generally, Bangladeshi men do not want to compromise on food-related issues [49]. The urban wife may be less justified for wife-beating because of burning foods and more significant socio-economic development. The urban people used technologically advanced cooking instruments than people lived in rural areas. Moreover, the difference in acceptance of wife-beating between rural and urban women can be traced in part to the influence of traditional norms and values, which persist in rural areas but are rapidly fading in urban settings due to the introduction of modernization components [51].

The findings reveal that age, education, marital status, number of children, socioeconomic status, mental or physical difficulties, problems getting pregnant, and husband’s age are the substantial factors correlated with the justification of wife-beating. The younger women who think of any one of the considered five reasons are less likely to be suffered from wife-beating than women aged more than 30 years [50, 52]. Typical reasons for this may be the increase of family size and financial needs with the duration of the marriage. However, financial status is not growing up accordingly to fulfil their expectations. In addition, the older aged women lost their husband’s attraction in the sense of attractiveness. The youngest males have a lower proclivity for violence against women. Therefore, programmatic interventions should transfer male attitudes regarding wife-beating when men are still young—before marriage [52].

Women with higher educational levels and social class justify wife-beating at a significantly lower rate than their counterparts, and these findings are supported by several previous studies [10, 22, 40, 50, 53]. In Bangladesh, higher educated women are more aware of their rights, and some of them are involved in jobs and business. Therefore, they have higher socioeconomic status and can contribute financially to their families [26]. On the other hand, when men are repeatedly unable to meet their families’ financial obligations, they can become frustrated and angered at the slightest provocation. As a result, they may consider using physical violence to reduce the financial strain [54]. In addition, other research has revealed a strong correlation between financial dependency and physical violence [55, 56]. This association recommends that socioeconomic inequalities and education be considered for planning intervention programs to empower women and lessen the justification of wife-beating. In developing countries like Bangladesh, mentally or physically challenged women are more likely to be justified for their caretaker’s wife-beating, e.g., partner or family member [57, 58]. Unable to involve in household works, financial issues, husband’s sexual demand, communication barriers, a lack of support structures, limited mobility, social exclusion, and negative social perceptions may increase the risk of wife-beating [58–60].

The residence is also a significant determinant for a greater acceptance of wife-beating in some circumstances in rural areas than urban areas [33, 35, 50]. Moreover, the results indicated that the women from the Barishal division were less likely to be okay justified with wife-beating than the Sylhet division, and women from the Rajshahi division had the highest odds (AOR: 2.42) of not justifying wife-beating among all divisions in urban areas. However, women from the divisions of Rangpur (AOR: 3.1), Khulna (AOR: 3.09), Chattogram (AOR: 2.74), Rajshahi (AOR: 2.52), Dhaka (AOR: 2.44), and other divisions were more likely to be justified for wife-beating if she thinks of any one of the considered five reasons, compared to Sylhet division in rural areas of Bangladesh. The authors think this variation is due to the demographic, cultural, and socioeconomic diversity within and between the divisions [61].

The findings of this study are based on the most recent and extensive nationally representative surveys (MICS-2019) of Bangladesh. Another potential strength of this study is that it is the first study to explore the factors associated with the justification of wife-beating in urban–rural settings of Bangladesh. However, the data in this study were collected at a single point in time, and we attempted to determine if there was a causal relationship between various characteristics and attitudes against wife-beating. Therefore, the authors think that this study should be conducted further by combining MICS data of previous years to check the long-term effect of identified influential factors. Hence, a mixed/qualitative approach is needed to get further insights..

Moreover, the existing literature supports the fact that IPV/wife-beating has increased dramatically during the COVID-19 pandemic; therefore, the findings of this study may be under-fitting to the ongoing pandemic duration [62–67]. The COVID-19 pandemic has accelerated all of the existing influential factors of wife-beating with new factors such as economic downturns, passing more time together at home, and pandemic induced fear, anxiety, depression, and stress. All of these factors contribute to intensifying intimate partner violence all over the world [68, 69]. Therefore, an investigation into this matter with a nationwide survey would produce some interesting insights.

## Conclusions

The research uses a large population-based sample with countrywide coverage and an ordinal logistic regression model to investigate the linkage between women’s socioeconomic characteristics and their justification for wife-beating. Women’s tolerance, i.e., the prevalence of wife-beating, differs according to where they live, their educational qualifications, whether they have any functional difficulties or are unable to conceive, their husband’s age, and their financial status. Therefore, it is necessary to take a proper action plan considering the education and occupation of women to enhance awareness of the distinct consequences of wife-beating in women, particularly in rural areas of Bangladesh. Finally, the authors believe that the findings of this research are essential for policymakers who want to empower women and eliminate the reason for wife-beating and IPV against women in Bangladesh.

## Data Availability

The datasets generated and/or analysed during the current study are available in the UNICEF Bangladesh repository. After registration, the data set is available via the following access link https://mics.unicef.org/surveys.

## Abbreviations

IPV: Intimate partner violence
LMIC: Low- and middle-income country
MICS: Multiple Indicator Cluster Survey
DV: Domestic violence
WHO: World health organization
UNICEF: United Nations Children’s Fund
EA: Enumeration area
OLR: Ordinal Logistic Regression
OR: Odds ratio
IBM: International Business Machines
SPSS: Statistical Package for the Social Sciences
AOR: Adjusted odds ratio
COVID-19: Coronavirus disease
